# Barcode clonal tracking of tissue-resident immune cells in rhesus macaque highlights distinct clonal distribution pattern of tissue NK cells

**DOI:** 10.3389/fimmu.2022.994498

**Published:** 2022-11-10

**Authors:** Chuanfeng Wu, Jialiu A. Liang, Jason M. Brenchley, Taehoon Shin, Xing Fan, Ryland D. Mortlock, Diana M. Abraham, David S.J. Allan, Marvin L. Thomas, So Gun Hong, Cynthia E. Dunbar

**Affiliations:** ^1^ Translational Stem Cell Biology Branch, National Heart, Lung, and Blood Institute, National Institutes of Health (NIH), Bethesda, MD, United States; ^2^ Barrier Immunity Section, Lab of Viral Diseases, National Institute of Allergy and Infectious Diseases (NIAID), National Institutes of Health (NIH), Bethesda, MD, United States; ^3^ Cellular and Molecular Therapeutics Branch, National Heart, Lung, and Blood Institute, National Institutes of Health (NIH), Bethesda, MD, United States; ^4^ Division of Veterinary Resources, Office of Research Services, National Institutes of Health, Bethesda, MD, United States

**Keywords:** barcode, rhesus macaque, tissue-resident, T cells, B cells, NK cells

## Abstract

Tissue resident (TR) immune cells play important roles in facilitating tissue homeostasis, coordinating immune responses against infections and tumors, and maintaining immunological memory. While studies have shown these cells are distinct phenotypically and functionally from cells found in the peripheral blood (PB), the clonal relationship between these populations across tissues has not been comprehensively studied in primates or humans. We utilized autologous transplantation of rhesus macaque hematopoietic stem and progenitor cells containing high diversity barcodes to track the clonal distribution of T, B, myeloid and natural killer (NK) cell populations across tissues, including liver, spleen, lung, and gastrointestinal (GI) tract, in comparison with PB longitudinally post-transplantation, in particular we focused on NK cells which do not contain endogenous clonal markers and have not been previously studied in this context. T cells demonstrated tissue-specific clonal expansions as expected, both overlapping and distinct from blood T cells. In contrast, B and myeloid cells showed a much more homogeneous clonal pattern across various tissues and the blood. The clonal distribution of TR NK was more heterogenous between individual animals. In some animals, as we have previously reported, we observed large PB clonal expansions in mature CD56-CD16+ NK cells. Notably, we found a separate set of highly expanded PB clones in CD16-CD56- (DN) NK subset that were also contributing to TR NK cells in all tissues examined, both in TR CD56-CD16+ and DN populations but absent in CD56^+^16^-^ TR NK across all tissues analyzed. Additionally, we observed sets of TR NK clones specific to individual tissues such as lung or GI tract and sets of TR NK clones shared across liver and spleen, distinct from other tissues. Combined with prior functional data that suggests NK memory is restricted to liver or other TR NK cells, these clonally expanded TR NK cells may be of interest for future investigation into NK cell tissue immunological memory, with implications for development of NK based immunotherapies and an understanding of NK memory.

## Introduction

Tissue resident (TR) immune cells play central roles in acting as “first responders” to microorganisms, local inflammation, trauma, or tumor initiation in local tissue environments (1). In addition, communication with surrounding cells or migration to other locations is critical to maintaining tissue homeostasis and robust adaptive and innate immune functions. Understanding the immune system in the context of both TR and circulating immune cells is essential for successful development of vaccines, cellular therapies, and other immunotherapeutic.

TR T cells have unique phenotype, function, and T cell receptor clonal patterns, even among T cells specific for the same epitope (2, 3). There is marked heterogeneity of T cells between tissues, among different individuals, and across ages (4–6). Most studies to date have focused on murine models or human disease samples. Non-human primate (NHP) models offer marked similarities to outbred populations of humans in terms of tissue-specific memory T cell responses to viral infections and serve as critical model organisms for viral pathogenesis and treatment investigations (7). Much of B cell humoral immunity develops and is maintained in secondary lymphoid tissues and bone marrow. Memory B cells are found in both blood and tissues (5, 8). Many methods have been developed to study antigen-specific B cells; however, most have focused on peripheral blood (PB) B cells, with less known regarding tissue B clonal repertoire, particularly in non-human primates and humans (9).

NK cells have important roles in both innate and adaptive immunity, tissue homeostasis, and are under current active development as immunotherapeutic (10–13). Recent high resolution immunophenotyping as well as single cell “omic” studies have revealed that human NK cells are highly heterogeneous in phenotype, developmental status, and functions across various tissues, individuals, and ages (14–16). A recent study in NHPs applied multi-omics analyses to study changes in tissue distribution, phenotype, and function in response to simian immunodeficiency virus infection (SIV) (17). In contrast to T and B cells, NK cells lack rearranged germ-line receptors able to serve as endogenous clonal markers, impeding understanding of NK cell pool maintenance and expansion, differentiation pathways linking various phenotypic and functional subsets, and relationships between tissue-resident and circulating populations. The diverse NK receptor repertoire includes combination of expressed activating and inhibitory NK receptors directly linked to specific NK phenotypes and functions in response to viral infections or other challenges (18).

Accumulating evidence for specific memory properties of certain subsets of tissue resident NK cells has been reported in the past decades. The G-protein coupled C-X-C motif chemokine receptor 6 (CXCR6) was reported to be required for NK memory in mice (19, 20), and liver-resident CXCR6^hi^ NK cells have adaptive immunologic memory properties in response to haptens or viruses in murine models (19, 21, 22). CXCR6+ NK cells are also enriched in human liver (23).Humanized murine and human studies suggest that human NK cells with a tissue-resident NK phenotype may mediate adaptive immune responses upon vaccination or infection (24). Splenic and hepatic NK cells with memory properties were also documented in non-human primates (25, 26). CD49a, an integrin involved in retention of cells in tissues *via* binding to collagen and laminin, has been identified as a tissue resident NK marker, and proposed as a marker of TR NK cells with memory properties (27, 28). DX5−CD49a+ NK are liver-resident as shown *via* parabiosis experiments in mice (20), and important for support of the maternal-fetal placental interfaces (29). NKG2C (30–33) and other cell surface and signaling proteins have been proposed as important in sustaining memory NK cells (34, 35). These advances in uncovering adaptive NK cell properties further stimulates interest in understanding NK clonal hierarches, particularly in tissue-resident populations, given that adoptive transfer of memory has conveyed by liver and spleen NK cells more readily than circulating NK cells in both murine and macaque models (21, 25, 26, 36).

Rhesus macaques (RM) NK cells are overall phenotypically and functionally similar to human NK cells, thus RM models have been employed to study NK cell biology, with high translational relevance. RM NK have been defined as CD3-CD14-CD20-NKG2A/C+ cells, with most RM NK cells co-expressing Nkp80 and CD8a (37–39). The available human anti-NKG2A antibodies recognize both activating NKG2C and inhibitory NKG2A receptor isoforms (37, 38, 40). Based on CD16 and CD56 expression, NKG2A/C+ NK cells in RM are classified into three distinct NK subsets: CD16+CD56- (CD16+), CD56+CD16- (CD56+) and CD56-CD16- (double negative or “DN”) (41, 42). RM CD56+ and CD16+ NK cells closely resemble human CD56^bright^ and CD56^dim^ NK cells, respectively (38, 43, 44). DN NK cells in RM have not yet been fully phenotypically and functionally characterized, but may represent an intermediate maturation step between CD56+ and CD16+ NK cells based on gene expression analyses in rhesus (43). In human, several potential intermediate NK cell populations have been reported, such as CD16+ CD56^bright^ cells (45), CCR7− CD56^bright^ cells (46), CD94^bright^ CD56^dim^ cells (47), and CD62L+ CD56^dim^ cells (48). The ontogeny of these human intermediate subsets is not well established. Using intravascular staining, we recently reported distinct trafficking dynamics for each RM NK cell population between blood and tissues (42).

Clonal tracking *via* introduction of high diversity genetic barcodes into hematopoietic stem and progenitor cells (HSPCs) allows direct insights into immune cell developmental relationships, population dynamics, and response to perturbations (49). We have utilized this approach in RMs to study the ontogeny of circulating NK cells, overcoming the lack of endogenous clonal markers in this cell lineage (41, 50, 51). In our barcoded RM model, we discovered killer Ig-like receptors (KIR)-restricted large clonal expansions of mature PB CD56-CD16+ NK cells, unexpectedly clonally distinct from myeloid, T cell, B cell and CD56+16- NK cells. These clonally expanded PB CD56-CD16+NK cells persist for months to years, often waxing and waning over time, suggesting self-renewal independent of ongoing production from HSPCs (41, 50).

In the current study, we now utilize our barcoded HSPC RM transplantation model to investigate the clonal characteristics of T, B, and NK cells across various RM tissues, and compare theses TR immune cells with their counterparts in the PB. Ultimately, our goal is to gain insights into the developmental pathways and tissue localization of these cell populations, with implications for their functions in specific microenvironments.

## Materials and methods

### Animals and autologous rhesus macaque barcoded HSPCs transplantation

Rhesus macaques (*Macaca mulatta*) (RMs) used in this study were housed and cared for by the NHLBI Non-human Primate Program. Animal protocols were reviewed and approved by the NIAID and NHLBI Division of Intramural Research Animal Care and Use Committees in Bethesda, MD (protocols H-0136 and LVD-26E). All procedures were carried out in accordance with institutional and national guidelines and laws governing research in animals including the Animal Welfare Act. The construction of barcoded lentiviral libraries; technical validation of their diversity and retrieval, and the RM barcoded HSPC autologous transplantation model have been previously described in detail by our group (50, 52, 53). In brief, RM CD34+ HSPCs were mobilized from the bone marrow by treatment with granulocyte colony-stimulating factor and plerixafor. Mononuclear cells were collected *via* apheresis, and CD34+ cells enriched by CD34+ immunoselection, and transduced with a high diversity lentiviral barcoded library, with diversity sufficient to ensure 95% or greater chance that each HSPC would be labeled by a unique barcode (50, 53). Following transduction, the autologous CD34+ HSPCs were infused intravenously back into the macaque conditioned with 1000 cGy total body irradiation.

### Tissue collection and processing

Mononuclear cells and granulocytes from the blood were purified using Ficoll-Paque PLUS (GE Healthcare) gradient separation. Lymph nodes were surgically removed from the axilla or groin. Spleen and liver samples were collected by laparoscopy and gut pinch biopsies were obtained *via* endoscopy. Additional tissues were collected from ZJ31 at necropsy. Lymph node and spleen samples were mechanically dissociated then filtered through a 100μm cell strainer to obtain single cell suspensions. Spleen mononuclear cells were further enriched by *via* Ficoll-Paque PLUS gradient separation. Liver, jejunum, colon, and lung samples were processed using gentleMACS C Tubes and gentleMACS Octo Dissociator with heaters (Miltenyi Biotec) and digested with 0.02 mg/ml liberase TL (Roche) and 10 units/1ml deoxyribonuclease (DNase) I (Roche) *via* a programed digestion protocol with intermediate spinning at 37°C for one hour. Cells were washed with phosphate buffered saline (PBS) and filtered through a 100μm cell strainer. Red blood lysis was performed with ACK RBC Lysis Buffer (Quality Biological) as needed. Bronchioalveolar lavage (BAL) to collect respiratory secretions containing lung-associated immune cells was performed by instillation of 150ml of saline into the lung *via* bronchoscopy followed by collection *via* suction and cell concentration *via* centrifugation. All cells were either immediately sorted or were cryopreserved in 10% dimethyl sulfoxide (DMSO) and 90% heat inactivated fetal bovine serum (FBS) and stored in liquid nitrogen.

### Flow cytometric sorting and analysis

**Figure 1:**
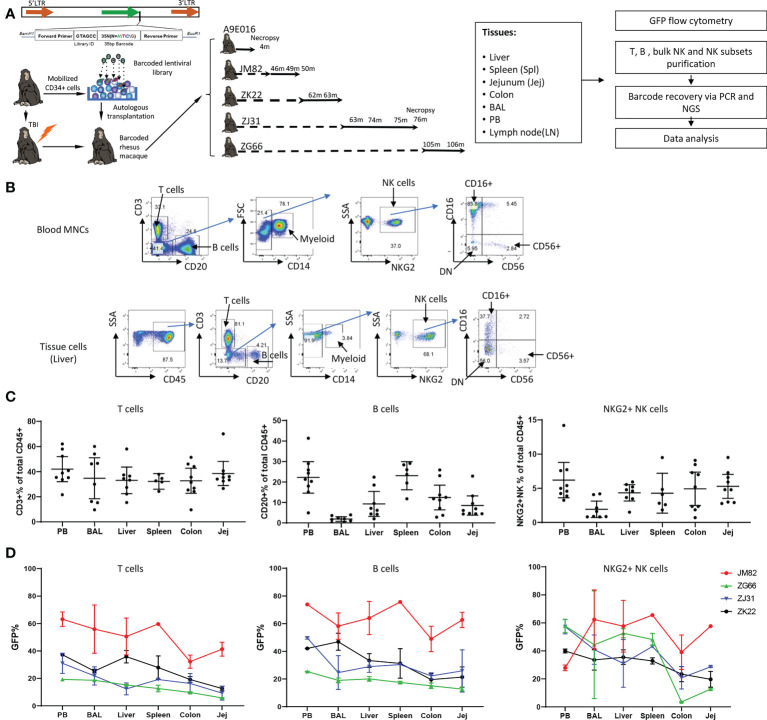
Experimental design, tissue and blood immune cell phenotyping and GFP marking levels **(A)** Experimental design. Peripheral blood (PB) and tissue samples including liver, spleen, jejunum, colon, lymph nodes and bronchoalveolar lavage (BAL) cells were collected at the specified time points post-transplantation from rhesus macaques transplanted with autologous CD34+ hematopoietic stem and progenitor cells (HSPCs) transduced with a high diversity barcoded lentiviral library. B cells, T cells, NK cells and NK cell subsets were analyzed for GFP expression and sorted *via* flow cytometry for barcode retrieval. **(B)** Representative flow cytometric gating strategies for purification of T cells, B cells, myeloid CD14+ cells and NK cells/NK cell subsets from PB (upper panel) and liver (lower panel). For all tissue samples, mononuclear cells were first gated on CD45+ cells before analysis and sorting based on hematopoietic lineage surface markers. **(C)** The percentage of CD3+ T cells (left panel), CD20+ B cells (middle panel) and CD3-CD20-CD14-NKG2+ NK cells (right panel) within the total CD45+ cells in tissues and mononuclear cells in PB. Jej-jejunum; Spl-spleen. All samples collected from animals from ZK22, JM82, ZJ31 and ZG66 are included. **(D)** The % GFP-expressing cells within CD3+ T cells (left panel), CD20+ B cells (middle panel) and CD3-CD20-CD14-NKG2+ NK cells (right panel) in PB and tissues (liver, spleen, colon, BAL and jejunum). Each line represents all data from an individual monkey as specified on right of each panel.

Fresh or thawed cryopreserved cells were stained with antibodies against lineage-defining cell surface markers (**Table S4**). Antibodies were mixed and added to the cells in 200ul PBS based FACS buffer with 2% BSA and 200mM EDTA, incubated 25 minutes at room temperature, washed with 3ml FACS buffer, and then resuspended in 400-800 μL FACS buffer for analysis or sorting. Flow cytometry analyses were performed using the BD LSRFortessa™ Cell Analyzer. Cells were sorted on a BD FACSAria II or a BD Symphony S6 sorter. Gating strategies are shown in **Figure 1B** and **Figure S1**.

### NK cells functional analyses

Healthy human donor PBMNCs or monkey PBMNCs were co-cultured without or with K562 cells (ATCC) in RPMI1640 medium with 10% FBS and 1x pen-strep antibiotics. Effector to target cells ratios of 5:1 or 10:1 were tested. After two hours target cell stimulation, PBMNCs were stained with Live/Dead viability dye, antibodies to CD3, CD14, CD20, NKG2A, CD16 and CD56, and the degranulation marker CD107a for flow cytometric analysis on the BD LSRFortessa™ Cell Analyzer. For cytokine analyses, PBMNCs were treated with IL-12 (10ng/ml), IL-15 (10ng/ml) and IL-18 (100ng/ml) overnight or stimulated with K562 at effector: target ratio at 5:1 or 10:1 for 1 hour, then monensin (Golgi-Stop, BD Biosciences) and brefeldin A (Golgi-Plug, BD Biosciences) were added per product instructions. 4-5 hours later, cells were washed and stained with Live/Dead viability dye, antibodies to CD3, CD14, CD20, NKG2A, CD16 and CD56. Cells were washed and then fixed with Cytofix/Cytoperm (BD Biosciences), followed with intracellular staining with anti-IFNγ and anti-TNFα mAb in PermWash buffer (BD Biosciences). Multi-parameter flow cytometry analysis was performed on BD LSRFortessa™ Cell Analyzer.

### Barcode recovery

Genomic DNA from > 200,000 cells was extracted with the DNeasy Kit (Qiagen). DNA from < 200,000 cells was processed using DirectPCR lysis buffer (Viagen Biotech). 200-500ng DNA or the whole cell lysis from each cell population was used for barcode retrieval. PCR amplification (28 cycles) of the barcode region was performed using a forward primer with a unique i5 index for each sample and a universal reverse primer (**Table S5**), using Phusion High-Fidelity DNA Polymerase (Thermo Fisher Scientific). After gel purification of the PCR products, 40-80 multiplexed samples were pooled for sequencing on an Illumina HiSeq 2500, 3000 or NovaSeq 6000 system to recover the barcodes (41, 50).

### Data analysis

Barcode sequencing fastq output files were processed using custom Python code to identify barcoded clones contributing above sequencing error and sampling thresholds (54, 55). Data analysis, Pearson correlations and plot generation were performed using R (Foundation for Statistical Computing) and Prism (GraphPad Software). Custom R code is available on GitHub at https://github.com/dunbarlabNIH/barcodetrackR and https://github.com/dunbarlabNIH/tissue_NK and the various analytical approaches and visualizations employed were described previously (54). Flow plots were generated by FlowJo V10 (FlowJo, LLC).

## Results

### Barcoded RM model for clonal tracking of immune cells

We previously utilized a barcoded HSPC autologous transplantation RM model to analyze the clonal relationships between blood lineages and over time, focusing on NK cells and other hematopoietic lineages (50, 55). We now apply the same approach to investigate clonal relationships across tissues in comparison to circulating immune cell populations. Briefly, a high diversity genetic barcode library was inserted into a lentiviral vector also expressing Cop green fluorescent protein (GFP). The barcoded viral library was used to transduce CD34+ HSPCs, introducing a unique barcode tag into each individual transduced cell. The transduced cells were infused into the autologous macaque following TBI conditioning. Following engraftment and hematopoietic recovery, hematopoietic lineage cells derived from the barcoded transduced HSPCs can be identified in PB and tissues *via* GFP expression, and clonally characterized *via* barcode retrieval. We collected concurrent PB, BAL fluid, and lung, liver, spleen, jejunum, and colon tissue samples from 5 barcoded monkeys (**Figure 1A**, transplantation parameters in **Table S1**). Cell populations were analyzed and sorted *via* flow cytometry (**Figure 1B**), followed by barcode retrieval from purified cell populations and quantitation of individual clonal contributions as previously described (50, 54, 55).

### Reconstitution of immune populations in tissues by transduced HSPCs

We first characterized lymphoid lineages within the CD45 hematopoietic compartment in tissues (**Figure 1B**). In PB as well as in all tissues, CD3+ T cells were most abundant and were relatively uniform across tissues, representing 35.9% ± 13.42% of hematopoietic cells in tissue samples from all animals and time points (**Figure 1C**). However, B cell abundance varied between different tissue sites: lowest in BAL and highest in spleen and PB, with intermediate contributions in liver and GI tract (jejunum and colon) (**Figure 1C**). In RM, blood and tissue NK cells can be defined as CD45+CD3-CD20-CD14-NKG2A/C+. In contrast to human NK surface markers, most RM CD16+ NK cells completely lose CD56 expression, and a significant population of double negative (DN: CD56-CD16-) NK cells can be found in blood and tissues (38, 39, 44, 56) (**Figure 1B**). NK abundance across animals and tissues varied, with overall somewhat lower percentages in tissues compared to PB (all tissues samples: 4.20% ± 2.51% vs PB: 6.21% ± 3.37%, mean ± SD, p=0.047) (**Figure 1C**).

The fraction of GFP expressing hematopoietic cells reflects reconstitution derived from autologous transduced HSPCs. We analyzed the frequency of GFP+ T, B and NK cells in PB and various tissues in four animals at time points 43-106 months post-transplantation (**Figure 1D**; **Figure S1**). The fraction of GFP+T cells in liver, spleen, and BAL was similar to PB, but the fraction in jejunum and colon was lower in each animal. This suggests that by these late time points post-transplantation, the T cell compartment in liver, spleen and lung has completely equilibrated with circulating HSPC-derived T cells; however, colon and jejunum immune tissue appears to retain endogenous TR T cells that are not replaced to the same extent by HSPC-derived T cells. The levels of GFP+ B cells were more uniform across tissues and well matched to concurrent PB, suggesting full reconstitution of the B cell compartment in these tissues.

Of note, the contributions of GFP+ NK cells were much more variable between PB and tissues, between different tissues, and even between different time points in the same tissues in individual monkeys (**Figure 1D**, **Table S2**). For example, JM82 had lower fractions of GFP+ NK cells in the PB compared to all tissues, ZG66 and ZJ31 had an overall higher GFP+ levels in PB compared to tissues, and ZK22 had quite similar levels in PB, spleen, liver and BAL compared to PB. In 3 (ZJ31, ZG66 and ZK22) out of 4 animals, the fraction of GFP+ NK cells in GI tract samples were lower than other tissues and blood. This heterogeneity, including over time and/or locations of biopsies in the same tissue, could be explained by differences in TR NK cell clonal behavior (i.e., a specific clone or set of clones expanding locally in a specific tissue site). We checked whether GFP expression could impact on NK cell function or phenotype. Our prior studies have not demonstrated impact on gene profile or lineage properties of macaque hematopoietic cells (57). We compared the phenotype and function of GFP+ versus GFP- NK cells from 4 RMs and found no significant differences in phenotypic distribution or activation/degranulation in response to K562 stimulation (**Figure S2**).

### Tissue-specific clonal expansions of T cells

**Figure 2:**
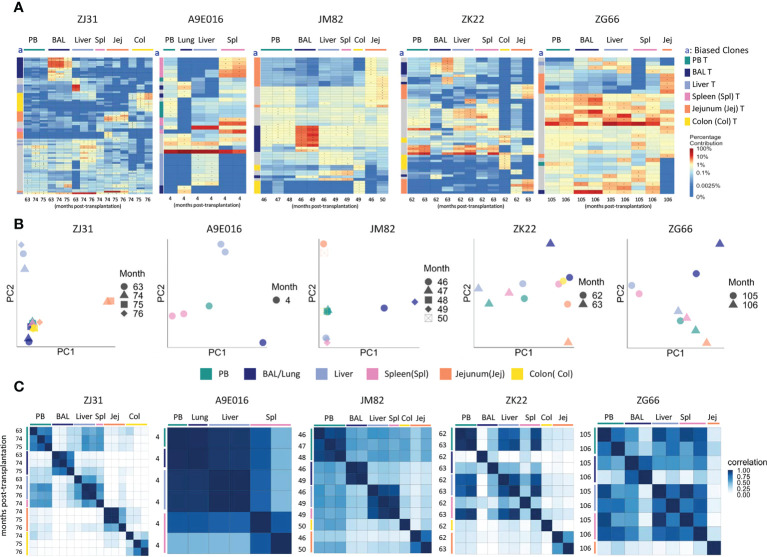
T cell clonality analyses in blood and tissues **(A)** Heat maps of the natural log fractional abundance of the highest contributing clones defined as all barcodes present as a top 10 highest contributing barcode in at least one of the T cell samples purified from PB or tissues, mapped over all samples for each animal (A9E016, JM82, ZK22, ZJ31 and ZG66). Each row corresponds to one unique barcode; * indicates the barcode is one of the top 10 for that sample. Each column corresponds to a tissue source and time point in months post-transplantation as labeled. Heat maps are organized by unsupervised hierarchical clustering of Euclidean distances between barcode’s log fractional abundances, with relative contributions for each clone specified per the color gradient shown on the far right. The column labeled “a” on the far left of each heatmap designate tissue-specific “biased” barcoded clones, defined as > 5x expanded in a specific tissue’s T cells compared to T cells from all other tissues or blood, ie clones that are expanded in one specific tissue compared to all other locations. For samples with multiple timepoints, the average clonal contribution across timepoints was used. Color coding for each specific set of biased clones is shown to the far right. Jej-jejunum; Spl-spleen; Col-Colon. **(B)** Principal component analysis (PCA) of the contributions from all T clones retrieved from PB and tissues. The samples collected from different anatomical locations are represented with different colors, and samples from the same location collected at different time points post-transplantation are denoted using different markers. For each sample, the barcode counts of all retrieved clones were first normalized to count-per-million (CPM). The normalized barcode counts were then transformed by projecting onto a lower dimensional space. The locations of the samples relative to each other on the PCA space indicates their degree of similarity. For example, in ZJ31, the jejunum samples across different timepoints cluster together but are located distantly from samples collected from other tissues. **(C)** Pairwise Pearson correlation coefficients heatmaps showing the pairwise clonal correlations of contributions from all barcodes retrieved from all PB and tissue T cell samples collected at the designated time points. The color scale for r values is shown on the right of the panels. Jej-jejunum; Spl-spleen; Col-Colon. A9E016 lung tissue was obtained at the necropsy at 4m instead of BAL collection.

Mature T cell clonal diversity and clonal expansions in PB and in some tissues have previously been studied *via* analysis of rearrangements of the T cell receptor (TCR) gene locus, primarily in mice and humans, with more limited information available for macaques (2, 58, 59). Quantitative genetic barcoding allowed us to study T cell clonal diversity, expansions, and relationships between circulating PB and TR T cells in RMs. Five RMs were transplanted at 3-6 years of age (young adult) and tissue samples and PB collected at 4 to 106 months post-transplantation for barcode retrieval. We focused on properties of the largest T cell clones contributing to any tissue or PB sample and tracked these clones over all samples for each animal to elucidate their geographic and longitudinal properties. Heat maps were organized by unsupervised hierarchical clustering of barcode abundances, with each row corresponding to a unique barcode, and each column to a specific sample (**Figure 2A**). In each macaque, unbiased clones showing similar contributions to tissues and PB T cells were detected (gray bars in column a, **Figure 2A**), suggesting that HSPC-derived T cell clones can seed tissues widely and continuously.

However, in addition to clones contributing homogeneously to various tissues and to PB, tissue-specific highly biased T cell clones were also detected in all animals as early as 4m post-transplantation (**Figure 2A**). “Biased” clones representing potential tissue-specific expansions were defined as showing at least 5-fold higher abundance in T cells from one tissue source compared to all other tissue and to PB and are designated by colored blocks next to the y axis of the heat maps (column a, **Figure 2A**). These biased clones were most apparent in BAL, jejunum, and colon samples. Of note, in animals with sampling performed from the same tissue at multiple time points within a 2–4-month period, the same tissue-specific expanded and biased T cell clones were detected at each time point, suggesting a stable tissue-specific T cell clonal repertoire maintained locally in specific tissues. This was true even in the intestinal sites, where the location of the biopsy along the intestine was completely different at the two time points sampled. This result was also confirmed for all clones (not just the largest clones visualized in heat maps) by principal component analysis (PCA) analysis (**Figure 2B**), where the normalized barcoded clone counts from all the samples were projected onto a lower dimensional space to help identify global patterns in clonal relationships between different tissue sites. In individual monkeys, the same tissues are closer in PCA space due to the very similar clonal pattern over time in the tissue. Pairwise Pearson correlation coefficients heatmaps (**Figure 2C**) visualize the clonal relationships considering both the clone numbers and the levels of each clone’s contribution to a sample. T cells from a specific tissue over time were highly corelated with each other but had lower correlations with T cells from the other tissue sites and PB (**Figure 2C**).

### Homogeneous clonal reconstitution of tissue myeloid and B cells

As previously reported, rhesus macaque PB B lineage and myeloid cells (granulocytes and monocytes) are initially derived from lineage-restricted short-term repopulating clones immediately following engraftment. By 3-6 months, these lineages shift to derivation from multipotent clones that contribute stably long term, with very limited degrees of B versus myeloid clonal bias (50, 55). We extended clonal analyses of these lineages to tissue sites.

**Figure 3:**
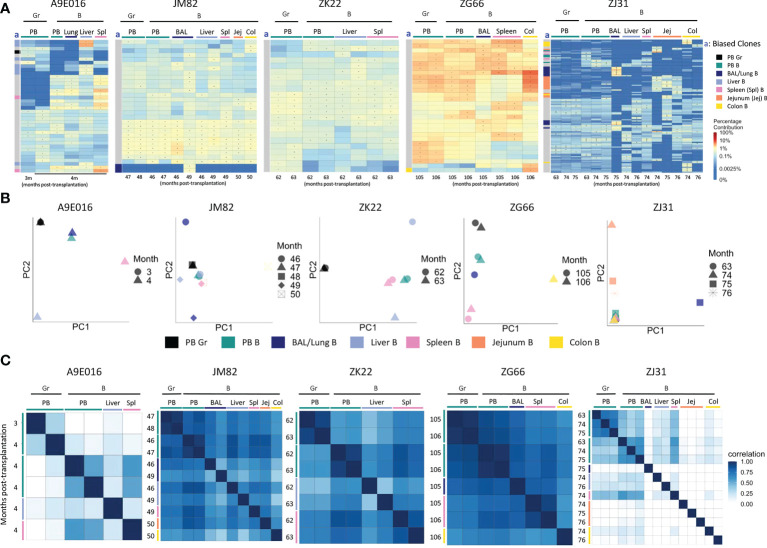
B cell and myeloid cell clonality analyses in blood and tissues **(A)** Heat maps of the natural log fractional abundance of the highest contributing clones defined as all barcodes present as a top 10 highest contributing barcode in at least one of the PB granulocyte (Gr), PB B cell or tissue B cell samples purified from tissues at the designated time points, mapped over all samples for each animal (A9E016, JM82, ZK22, ZJ31 and ZG66). Heatmaps were constructed as in **Figure 2A**. The column labeled “a” on the far left of each heatmap designate tissue-specific “biased” barcoded clones, defined as > 5x expanded in a specific tissue’s B cells compared to B cells from all other tissues or Gr from blood, ie clones that are expanded in one specific tissue compared to all other locations. For samples with multiple timepoints, the average clonal contribution across timepoints was used. Color coding for each specific set of biased clones is shown to the far right. Jej-jejunum; Spl-spleen; Col-Colon. **(B)** PCA analysis of the Gr and B cell clones from PB and B cell clones from tissues. The samples collected from different anatomical locations are represented with different colors (color legend located below the PCA plots), and samples from the same location collected at different time points post-transplantation are denoted using different markers. PCA projections are performed as detailed in **Figure 2B**. **(C)** Pairwise Pearson correlation coefficients heatmaps showing the pairwise clonal correlations of contributions from all barcodes retrieved from PB Gr and B cells and from tissue B cells collected at the designated time points post-transplantation. The color scale for r values is shown on the right of the panels. Jej, jejunum; Spl, spleen; Col, Colon. * in the barcode heatmap indicates the barcode is one of the top 10 for that sample.

Animal A9E016 had tissues collected during a planned autopsy at 4m. As shown by both top clones heatmaps and PCA analyses, the PB contained output from both shared B/myeloid clones and myeloid or B cell biased clones, demonstrating that the transition to B and myeloid output from multipotent HSPC was not yet complete at this early time point (**Figures 3A, B**). Liver and spleen contained both tissue-biased B cells, as well as B cells derived from clones also contributing in the PB. In JM82, ZK22 and ZG66, tissue sampling occurred years following transplantation, when B cells and granulocytes in the PB were derived primarily from multipotent stable unbiased clones. In these animals, tissue B cells were closely related to PB B cells and granulocytes, as well as to B cells in other tissues. This observation is confirmed by top clone analyses, PCA of all clonal contributions, and Pearson pairwise correlations (**Figures 3A–C**). There were very few biased clones found with >5-fold abundance in one tissue versus PB or other tissues (**Figure 3A**), and PB and tissue samples generally clustered together in PCA space in these 3 animals. In addition to shared clones, samples from ZJ31 obtained at these late time points showed some tissue B cell clones that appeared highly biased towards contributions to only BAL, colon, or jejunum samples respectively. However, a caveat is that each HSPC clone in ZJ31, an extremely polyclonal animal with thousands of HSPC clones, contributes at a very low level, potentially resulting in artifactual estimates of clone size due to constraints resulting from analyses of lower available B cells analyzed from jejunum, colon, and BAL samples (**Table S3**). However, the retrieval of this same set of jejunum-biased B clones from samples collected across three different time points/sampling locations in jejunum does suggest this finding is not artifactual and provides evidence for accumulation and expansion or selective persistence of B cell clones in certain tissues, presumably in reaction to local stimuli by these clones specifically.

### NK subsets in tissues and blood

**Figure 4:**
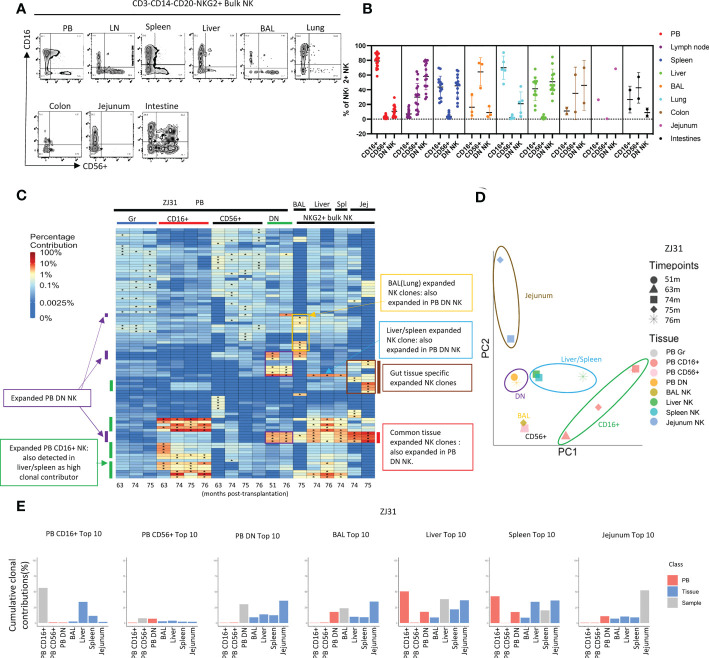
Natural killer cell clonality analyses in blood and tissues **(A)** Representative flow cytometric analysis of CD16 and CD56 expression within PB and tissue NKG2+ NK cells. **(B)** Summary of the distribution of NKG2+ NK cell subsets based on CD16 and CD56 expression in blood and tissues. DN- “double negative” for both CD16 and CD56 expression. Individual samples shown as individual dots. **(C)** Heat maps of the natural log fractional abundance of the highest contributing clones defined as all barcodes present as a top 10 highest contributing barcode in at least one of the PB granulocyte (Gr), PB NK cell subsets, or tissue bulk NKG2A+ NK cells purified from tissues collected from animal ZJ31 at the designated time points, mapped over all samples for the animal. Heatmaps were constructed as in **Figure 2A**. The biased/expanded PB NK subset clones were defined as 5 folds expanded in a specific PB NK subset compared to other PB NK subsets and PB Gr. The tissue specific biased/expanded NK clones were defined as 5 folds expanded in a specific tissue NK population compared to all other NK populations in other locations. Jej-jejunum; Spl-spleen. **(D)** PCA analysis of the Gr and NK subset clones from PB and bulk NK clones from tissues. The samples collected from different anatomical locations are represented with different colors, and samples from the same location collected at different time points are denoted using different markers. The PCA projections were performed as detailed in **Figure 2B**. **(E)** The % clonal contributions of the aggregate top 10 clones from one NK population to all other populations (i.e., comparing the contributions of the top 10 clones from PB CD16+ NK to other PB NK subsets and to tissue NK cells from various locations). The largest 10 clones for each PB NK subset or tissue NK source were based on normalized contributions of clones over all time points available for that sample type from the animal. In each bar plot, the contribution of the aggregate top 10 clones of the sample to itself is shown in grey, the contribution of the same clones to PB NK subsets is shown in red, and the contribution to the same clones to tissue NK populations is shown in blue. * in the barcode heatmap indicates the barcode is one of the top 10 for that sample.

Tissue or PB NK cells were defined as CD3-CD14-CD20-NKG2+, and NK subsets were further sorted based on CD16 and CD56 expression. The phenotype of the CD56/CD16 NK subsets in tissues varied (**Figures 4A, B**), with the fraction of CD16+ NK highest in PB and lowest in BAL and LN. Conversely, BAL and LN contain the largest fractions of CD56+ NKs. DN NKs can be found at high levels in liver, spleen, and LN (**Figures 4A, B**). Extended phenotyping of the NK subsets in the different tissues was performed (**Figure S3**), focusing on the chemokine receptors CX3CR1 and CXCR3 previously linked to NK tissue infiltration and migration, the activation markers CD69 and NKG2D, the putative tissue-resident adaptive NK marker CD49a, and the tumor necrosis factor receptor superfamily member CD27 which has been reported as a memory NK marker in mice and heterogeneously expressed across human NK subsets (60) (61). CX3CR1 and CD69 expression was most frequent were higher in CD16+ tissue NK compared CD56+ and DN NK. CXCR3 levels were highest in liver NK compared to other tissues. NKG2D levels varied across different NK subsets and tissues, highest in LN, liver, and spleen and lowest in PB. CD49a was found on CD56+ and DN NK subsets in all tissues. In contrast, few CD16+ NK in PB expresses CD49a, but LN, liver, and spleen CD16+ NK expressed CD49a more frequently than other tissue CD16+ NK. CD27 expression were detected in all NK subsets in PB at a higher frequency than in tissue NK, particularly liver NK subsets do not express CD27. Differences in these NK phenotypes between locations may result from variable ontogeny, maturation stages and/or function.

To further investigate the functionality of the various RM NK subsets, we performed degranulation and cytokine production analyses on NK from the RMs, comparing to normal human NK cells, focusing on CD16+ NK, CD56+ NK and DN NK subsets in RM samples, CD56^dim^, CD56^bright^, and CD56^dim^CD16- NK in human samples (**Figures S4**, **S5**). In RM NK, CD16+ and DN cells express similar levels of surface CD107a post K562 stimulation, much higher than CD56+ NK, suggesting both the CD16+ and DN NK have high killing potential. In human NK, all three subsets have degranulation potential, interestingly CD56^dim^CD16- have the highest level of CD107a in response to the target cells. The highest frequency of RM CD56+ cells produced TNFα and IFNγ compared to CD16+ and DN NK cells. In human NK, CD56^bright^ most frequently produced IFNγ compared to CD56^dim^ and CD56^dim^CD16- NK subsets. In contrast to RM NK subsets, all human NK subsets produced IFNγ much more frequently than TNFα post cytokine treatment. Both human and RM NK cells produce primarily TNFα after K562 stimulation. These functional analyses reveal that RM DN NK cells have both killing and cytokine production capacities and suggest that they could be an intermediate functional population between CD56+ and CD16+ NK subsets, perhaps analogous to the CD56^dim^CD16-NK subset in humans, which also have both killing and cytokine capabilities, but further comprehensive phenotyping multi-omics profiling are needed.

### Shared clonal expansions between multiple tissue locations and PB DN NK cells

We compared clonal patterns between various TR and PB NK cells. Tissue NKG2+ NK clonality was initially analyzed in bulk rather than in NK subpopulations due to low cell numbers obtained from serial tissue biopsies, and possible changes in CD16 or CD56 expression in TR NK versus PB. In rhesus ZJ31, we first focused on the 10 highest contributing clones in each cell population, visualized by level of clonal contributions across all samples (**Figure 4C**). In PB, in addition to the previously reported highly expanded and lineage biased CD16+ NK clones (green bars on **Figure 4C**), an additional group of PB expanded/lineage biased DN NK clones were detected (**Figure 4C**, purple bars). These expanded DN NK clones had not been expanded in PB samples from earlier time points based on continuous data collected from 1m post transplantation to until 76m (**Figure S7**). These PB DN NK lineage-biased expansions were also detected in the other 3 monkeys, even in ZK22, an animal without markedly expanded CD16+ PB clones (**Figure S8A**). On PCA analysis of the level of all clonal contributions to each sample, PB DN NK do not cluster with PB CD16+ or CD56+NK (**Figure 4D** and **Figure S8B**). These results suggest a clonally distinct PB DN NK population, potentially responding to different stimuli or with different clonal expansion pathways as compared to CD16+ NK cells.

Interestingly, in ZJ31, many of the largest expanded PB DN clones were also expanded in tissues (red bars, **Figure 4C**). Furthermore, a PB DN expanded clone was also markedly expanded in liver and spleen, but not in gut or BAL (blue bars, **Figure 4C**). The differences in patterns of these clones within tissues suggests blood contamination cannot account for their presence (as well as the differences noted above in T cell contributions between tissues and blood). Shared high contributing clones found in both PB DN and tissue bulk NK were detected in the other 3 animal (**Figure S8A**, red bars), but the degree of expansions varied between monkeys and different tissue sites. For example, the two expanded PB DN clones in JM82 are also expanded in liver, but not in BAL. In ZK22, highly expanded PB DN clones are also expanded in the liver and spleen, but not to the same degree as in PB DN NK. In ZG66, two clones expanded in both CD16+ and DN NK in PB are also expanded in tissues including BAL, liver and spleen (**Figure S8A**).

As another measure of the relevance and relative size of PB NK clones in tissues, we compared the aggregated contributions of the top 10 clones PB NK clones from each subset to other PB NK subsets and to TR NK cells (**Figure 4E** and **Figure S8C**). As previously reported (41, 50), the aggregate top 10 PB CD16+ NK clones make up a very large fraction of PB CD16+ NK barcoded population overall in the four monkeys (43.3% ± 23%). In the tissues, however, these top PB CD16+ NK clones contribute at lower levels. The aggregate top 10 CD16+ NK clones had the highest contributions to the liver and/or spleen, this may be due to CD16+ NK being higher in liver and spleen than in other tissues (**Figure 4B**). PB CD56+ NK were highly diverse with many small clones present (**Figure S6**) (41). The aggregated top 10 PB CD56+ clones make up a relatively small proportion of the PB CD56+ NK subset in ZJ31, ZK22 and JM82. These clones show low contributions in tissues, indicating the largest PB CD56+ NK clones are not the major contributors to TR NK cells. However, the top 10 CD56+ clones make up to 36.5% to the PB CD56+ in ZG66, and those clones are also highly present in other NK subsets, tissue NKs, and PB myeloid granulocytes. Taken together, this indicates that the ZG66 top 10 CD56+ clones are not expanded in NK cells specifically but result from robust HSPC output from several larger HSPC clones in this animal.

In all four monkeys, the aggregate top 10 PB DN NK clones make up a very significant portion of all barcoded NK contributions in PB DN NK (given the 1000s of HSPC clones engrafted in each animal (55)) (37.14% ± 13.4%, mean±S.D), consistent with our observation of highly expanded clones within the PB DN NK lineage. Moreover, the clonal contributions of these top PB DN NK clones to the other two PB NK subsets were minor in ZJ31, ZK22 and JM82 (**Figure S8C**), except for the top 10 clones from PB DN NK in ZG66, also high contributors in other PB NK subsets and myeloid cells as detailed above (**Figure S8C**). As expected from the heat maps, the aggregated contributions of the top 10 DN NK to all tissues sampled were appreciable in ZJ31 and variable across the other animals. PB CD16+ NK top 10 clones contributed major fractions to liver and spleen NK cells. The variations in NK clonal patterns between tissues in different macaques may reflect the heterogeneity of clonal immune responses in individual animals depending on underlying genetic background or environmental exposures.

### Tissue-specific clonal expansions of NK cells

We next focused on sets of expanded clones found in tissues but not in PB. In ZJ31, we noted NK clones highly expanded within some tissues but not others. As shown in **Figure 4C**, we identified BAL/lung-specific (yellow box) and GI tract-specific (brown bar) tissue-biased expanded NK clones. In JM82, we observed BAL specific NK cells (**Figure S8A**). In ZG66 and ZK22 no tissue-specific NK clones were observed at the 5-fold expansion threshold, which could be attributed to the observations that in ZG66 and ZK22 tissue NK clones represent multipotent HSPC clones and these animals have fewer expanded NK clones in tissues (**Figure S8A**). Within individual animals, some tissue specific NK clones were consistently retrieved at time points from the same tissue, and these samples clustered together on PCA space (**Figure 4D** and **Figure S8B**), indicating the clonal stability in a specific tissue over at least a 1-to-4-month time period. Given that the exact site sampled in each tissue varied between time points, it supports a tissue-specific clonal reaction or clones that specifically home to a tissue. In macaques ZJ31, ZK22 and ZG66, with samples available from both liver and spleen, the liver and spleen NK clonal patterns were highly correlated, locating near each other in PCA space (**Figures 4C, D** and **Figures S8A, B**).

When we analyzed the aggregated contributions of the top 10 clones in each tissue across other tissues and PB, contribution patterns varied between monkeys. No common pattern was found, except that liver and spleen NK cells share most of their top 10 clones (**Figures 4C, E** and **Figures S8A, C**). Consequently, the shared liver and spleen NK clones led to similar aggregated clonal contributions pattern when we tracked between spleen and liver samples in each monkey (**Figure 4E** and **Figure S8C**).

### The clonal relationships between PB NK subsets and specific tissue NK subsets

**Figure 5:**
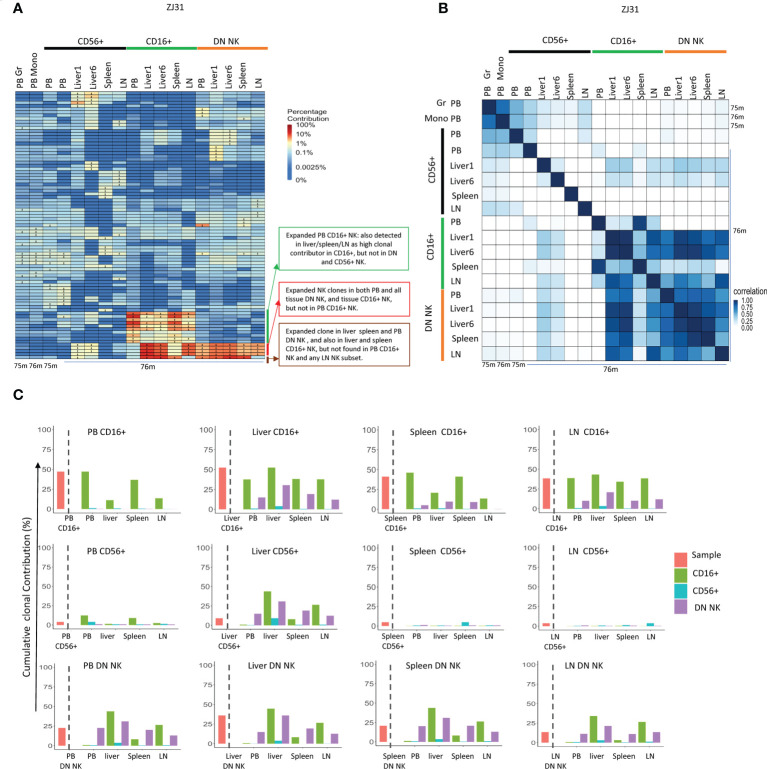
Tissue NK subset clonality analyses **(A)** Heat maps of the natural log fractional abundance of the highest contributing clones defined as all barcodes present as a top 10 highest contributing barcode in at least one of the PB granulocytes, monocytes or PB or tissue NK cell subpopulations in animal ZJ31. Samples were collected from ZJ31 at necropsy performed 76 months post-transplantation. Heatmaps were constructed as in **Figure 2A**. **(B)** Pairwise Pearson correlation coefficients heatmaps showing the pairwise clonal correlations of contributions from all barcodes retrieved from PB granulocytes, monocytes and PB and tissue NK cell subsets at 76 months post-transplantation. The color scale for r values is shown on the right of the panel. **(C)** The clonal contributions of the aggregate top 10 clones from one NK population to all other NK populations (calculated as detailed in **Figure 4E**). The schematic panel on the left diagrams how each panel is organized, with the contribution of the aggregate top 10 clones from the index sample to itself shown to the left of the dotted line, and to other NK populations to the right of the line, organized by tissue and by NK cell subset. * in the barcode heatmap indicates the barcode is one of the top 10 for that sample.

ZJ31 underwent euthanasia and autopsy due to discovery of a localized but large hepatocellular carcinoma. This gave us the opportunity to obtain sufficient material for sorting of NK subsets from each tissue (in contrast to the other macaques that are being followed on a long-term observation protocol). We purified separate NK cells from both normal liver lobes (liver 1) and from the tumor region. The clonal pattern of each NK subset from the different liver samples was similar, suggesting the clonal expansion in liver is homogenous across the tissue even in the presence of a localized tumor (**Figures 5A, B**).

TR CD56+ NK cells were polyclonal and without large expansions, overall matching myeloid and PB CD56+ clonality (**Figure 5A**). However, the clonal repertoire of CD56+ in some samples may have been underestimated due the low cell number present in some tissues such as liver with very few CD56+ NK cells. The highly expanded PB CD16+ NK clones were also expanded in tissues, particularly in liver and spleen, suggesting that similar factors may result in clonal expansions in this CD16+ NK subset in both tissues and PB or shared trafficking patterns. More interestingly, some CD16+ NK clones highly expanded in liver, LN and spleen were barely detected in PB CD16+. Instead, these clones matched those expanded in DN NK in tissues and in PB DN NK. DN NKs in PB and all tissues have similar clonal patterns and were highly correlated with each other (**Figure 5B**), except for one clone that was expanded in PB, liver and spleen DN but not found contributing to the LN (**Figure 5A**).

In terms of overall aggregate contributions, the PB CD16+ top clones contributed significantly only to PB and tissue CD16+ NK, but not to other NK subsets in any location. In contrast, the tissue CD16+ NK top 10 clones also made large overall contributions to DN NK in tissues and PB, suggesting a direct developmental relationship between these two NK subsets in tissues (**Figure 5C**). The PB DN NK top clones contributed significantly to tissue CD16+ and DN NK but not to CD56+ NK. Similarly, the tissue DN NK top clones contributed at a high level to DN and CD16+ NK in tissues but not to tissue CD56+ NK. CD56+ NK top clones did not make high contributions to any other NK subset, except for the liver. The liver CD56+ NK top clones contributed to tissue DN, tissue CD16+ NKs, and PB DN, but did not contribute to PB CD56+ and PB CD16+, suggesting possible unique CD56+ NK cell lineage derivation in the liver (**Figure 5C**).

CD49a was reported as putative marker for liver TR memory-like NK cells in humans (27, 28). In ZJ31, sorting for CD49a+ NK cells enriched for the large, expanded NK clones (**Figure S9A**, red bar) compared to CD49a- NK. However, we did not find that CD49a expression enriched for expanded TR NK cells in other locations such as the jejunum (**Figure S9**).

## Discussion

In this study, we analyzed immune cell clonality across various tissues in a barcoded HSPC rhesus macaque transplantation model, concentrating on the NK lineage, cells with characteristics of both innate and adaptive immunity but no endogenous clonal markers, in contrast to T and B cells. *Via* acquisition of serial lymphoid and non-lymphoid tissue biopsies, we were able to study the distribution, phenotype and clonality of these TR cells and make comparisons with circulating cell populations. This approach allowed us to identify tissue-specific clonal distributions and relationships of immune cells, including novel findings regarding NK cell subsets.

Prior detailed multiparameter immunophenotyping and transcriptional profiling of human NK cells in blood and tissues primarily uncovered heterogeneity within the CD56^bright^ subpopulation, particularly within tissue-resident populations in secondary lymphoid tissues and the gut, corresponding to both putative developmental stages as well as tissue-specific localization signals (16, 62). Tissue specific NK cell gene expression patterns have also been reported in murine single cell RNASeq studies (63). However, most of these studies have had more difficulty distinguishing distinct clusters within CD56^dim^ human NK cells, whether in blood or tissues, other than terminal differentiation or exhaustion markers such as CD57 and pan-adaptive markers such as NKG2C.

In PB, besides the expanded CD16+ NK clones, we observed highly expanded DN NK clones in all animals analyzed, distinct from the CD16+ expanded clones. DN NK cells have been previously demonstrated to represent a distinct functional subtype of macaque NK cells, showing “dual” functions of both robust interferon-γ secretion and cytotoxicity (44), generally associated with CD56+ macaque NK (or CD56^bright^ human) or CD16+ macaque (CD56^dim^ human) respectively. We confirmed these findings for DN NK cells from our animals. These cells thus may represent an intermediate population able to differentiate to CD16+ NK cells in tissues in response to specific stimuli, potentially corresponding to intermediate CD56^dim^ NK cells described in humans (64). We did observe shared clones between DN NK and CD16 NK populations in tissues, but not generally in blood. Of note, following SIV infection macaques increase the total number of DN NK cells, and upregulate expression of the gut lymphoid homing integrin α4β7 on these DN NK (65). These findings suggest that DN NK clonal expansion may play a role in RM response to pathogens and occur independently of the PB CD16+ compartment. The expanded DN NK clones detected in multiple tissues and in PB were long lasting (at least two years as shown in **Figure 4C**), possibly defining a distinct memory compartment of TR NK clones, not overlapping with CD16+ circulating putative memory NK cells.

Most notably, we observed sets of PB DN expanded clones that were also present and clonally expanded across multiple tissues in some animals. The sharing of these clones across multiple sites suggests preferential hematogenous homing of these clones to multiple tissues, with implications for the development of NK based immunotherapies and memory properties within this DN NK subset. Some of these same clones were also found expanded in tissue CD16+ NK cells, but not PB CD16+ NK cells, suggesting a possible developmental/maturation pathway between DN NK and CD16+ NK in tissues. It is also possible that PB DN NKs represent a linear developmental precursor to tissue resident DN and CD16+ NK cells which spend their remaining lifetimes in tissue but are not direct precursors for PB CD16+ NK cells.

In addition, sets of NK expanded clones specific to only a single tissues or tissues were detected, for instance in BAL or GI tract, or most strikingly clones found localized only to liver and spleen. In humans, NK cells with the tissue localization markers CD69+, CD103+ and/or CD49a+ were observed in lung tissue following influenza A infection. These populations made up 15-25% of lung NK cells, suggesting tissue-specific and/or pathogen-specific TR NK dynamics in the lung (66–69). The gut mucosal immune system functions as a critical front line innate immune response. Human studies have suggested that NK cell distribution and phenotype in the gut alters during HIV or human Herpesvirus 6 (HHV-6) infections (70–72). NK tissue diversity previously could only be accessed *via* immunophenotyping or single cell RNA profiles, without insights into clonality (16, 68, 73). In our study, we detected specific clonal expansions in BAL and gut tissue in RMs, with no or very low contribution to other tissues and PB, providing direct evidence for respiratory and gut specific NK responses related to tissue specific microenvironmental cues, and perhaps contributing to pathogen-specific memory in relevant barrier locations. Our observations of tissue-associated clonal NK cell expansions are particularly notable given prior adoptive transfer and functional studies in mice and primates linking memory properties to NK cells localized to liver and/or spleen instead of those circulating in the blood (19, 21, 22, 25, 74). A recent study reported a unique population of SIV-infected macaque PB NK NKG2^hi^CD16- cells which has upregulated genes associated with tissue homing (CXCR3), proliferation (Ki67), and transcription factors involved in cellular stemness and quiescence such as Tcf7 (TCF-1), indicating this population is at a low differentiation stage when compared to the other subsets based on NKG2 and CD16 expression (75). However, those NKG2^hi^CD16- cells did not resemble adaptive NK cells based on their gene expression profile. But the NKG2^low^CD16- NK cells displayed several adaptive NK cell-like features including low expression of *FcεRγ* and *VAV1* (75). *NKG2C* expression levels in all the NK sublets was low indicating this NK marker has very limited role in SIV as compared to CMV infection (75).

We demonstrated groups of balanced/un-biased CD56+ NK clones and DN NK clones contributing equivalently in PB CD56+ NK compared to tissues, suggesting a shared clonal repertoire between those populations. We recently utilized serial intravascular staining (SIVS) with multi-color labeled CD45 antibodies immediately binding to circulating blood cells to study movement of blood immune cells in and out of tissues (42, 76). We found that PB CD56+ and DN NK cells have a shorter residence time in blood and traffic more often between PB and tissues such as lymph nodes compared to the CD16+ NK subset, which remains primarily within the circulation. The rapid exchange between blood and tissue for CD56+ and DN NK cells may help explain the close clonal correlations between compartments for these subtypes compared to CD16+ NK cells.

In this study, we used an anti-NKG2A antibody, which recognizes both NKG2A and NKG2C isoforms in rhesus macaques, for bulk NKG2+ NK purifications from tissue and PB to select mostly NK cells. Multiple publications have also used this antibody to distinguish NKG2+ NK from NKp44+ type 3 innate lymphoid cells (ILC3) (77–80). However, human ILC can acquire CD94 (a protein that dimerizes with NKG2 receptors) expression *in vitro* (81), and recent publications have described subsets of human CD94+ ILC (82, 83). The natural cytotoxicity receptors (NCRs) NKp46 and NKp44 have been found expressed on both NK cells and ILCs (84). As noted previously (85), phenotypic separation or even definition of helper ILC1 and NK cells is problematic due to overlapping markers including CD127 and CD94 used as ILC/NK distinguishing markers for studies of human or macaque cells (86, 87). We and others have shown that CD127 and CD117 are expressed at low levels on human CD56^bright^ NK cells (88). Taken together, tissue NK and ILCs share several markers, and definitive markers to distinguish NK from ILCs in tissues remain unresolved in both human and NHPs, therefore we are not able to exclude the presence of ILCs in the bulk NKG2+ populations contributing to clonality.

Confirming prior TCR-based clonality studies and thus the validity of our clonal tracking approach, specific sets of T cell clones were found localized to various tissues in our macaque studies, suggesting local tissue T expansion and maturation related to location-dependent cues. Or the results may reflect the tissue competence imprinting of T cells development, by which tissue-derived migratory dendritic cells imprint tissue-specific homing patterns to maintain tissue specific T clones, as disscussed previously (89). Prior human studies have documented multiple tissue specific or tissue restricted memory T cells, with specific distribution of clones defined *via* TCR within T cell subsets (59). Previous studies have also reported tissue specific B cells and plasma cell clonal localization (5, 90), especially in gut (91) and lung (92). In our model, B cell clonality generally closely mirrored blood, with circulating B cells clones derived from ongoing active HSPCs, similar to short-lived granulocytes. However, in animal ZJ31, a group of both jejumun-specific and BAL-specific B clones were observed over time, evidence for tissue-specific B cell clonal expansions and selection in certain tissues in reaction to local stimuli, congruent with prior literature.

Our study and findings have several caveats. We analyzed primarily samples obtained more than 4 years post-transplantation, with only a single set of samples collected very early (4 months) and none at intervening time points. Thus, the time course for establishment of the NK clonal patterns observed cannot be defined. Restrictions on the frequency and absolute number of surgeries, as well as number and size of biopsies from live animals limited longitudinal data and the numbers of the cells collected at each time point. The clonal repertoire of thus may be underestimated, since small clones present in a cell sample may not be retrieved due to sampling constraints. Our focus on the top contributing clones to understand clonal relationships between tissues and cell types partly mitigates this issue. That tissue specific NK clones were able to be detected multiple times on serial biopsies from the same tissue suggests that our samples do reflect the tissue’s overall clonal pattern, at least for the high contributing clones. In addition, all tissues studied are highly vascularized and thus tissue biopsies may contain PB contamination, especially in the liver and spleen. Therefore, partial overlap between NK clones observed in PB and tissue may be from PB contamination, especially clones detected in the spleen and liver. However, our previous studies using intravascular staining to follow immune cell dynamics in macaques (42, 76) found very limited circulating CD45+ hematopoietic cells contaminating gut and LN biopsies. Our phenotypic analyses of NK cells in different tissues and PB revealed marked differences, even for liver or spleen, indicating the cells we obtained from all tissues have limited PB contamination.

Our model could be further utilized to study clonal changes in tissues and blood in response to infections, vaccines, or immunotherapies relevant to humans, particularly in the NK lineage. Further analyses of gene expression, epigenetic profiles, and clonal dynamics *via* utilization of a lineage tracing vector that permits barcode retrieval simultaneously with single cell gene expression and ATACseq analyses are ongoing and should allow insights into pathways driving clonal expansions in both tissues and blood, and developmental relationships between clones in various phenotypic populations in both blood and tissues. It will be of particular interest to investigate clonality and relationship in decidual NK cells, a uniquely functional NK population that we have not yet investigated at a clonal level (93).

## Data availability statement

The original contributions presented in the study are publicly available. This data can be found here: https://github.com/dunbarlabNIH/tissue_NK/tree/main/Data.

## Ethics statement

Animal protocols were reviewed and approved by the NIAID and NHLBI Division of Intramural Research Animal Care and Use Committees in Bethesda, MD (protocols H-0136 and LVD-26E).

## Author contributions

Conceptualization: CD, CW, DA, JB. Methodology: CW, DA, JL, RM, DA, TS, XF, MLT, SH. Investigation: CW, DA, JL, RM, DA, TS, XF, SH. Formal analysis: CW, JL. Visualization: CW, JL. Supervision: CD, CW. Writing – original draft: CW, JL, CD. Writing – review & editing: CW, JL, DA, CD. All authors contributed to the article and approved the submitted version.

## Funding

All work was supported by the intramural research programs of the National Heart, Lung and Blood Institute and the National Institute of Allergy and Infectious Diseases.

## Acknowledgments

The authors would like to thank Richard Herbert from NIAID, and Nathaniel Linde, Allen Krouse, Theresa Engels, and Justin Golomb from NHLBI for animal care and tissue retrieval, and Stefan Cordes for valuable discussions. The authors also acknowledge the contributions from the NHLBI Flow Cytometry and DNA Sequencing and Genomics cores.

## Conflict of interest

The authors declare that the research was conducted in the absence of any commercial or financial relationships that could be construed as a potential conflict of interest.

## Publisher’s note

All claims expressed in this article are solely those of the authors and do not necessarily represent those of their affiliated organizations, or those of the publisher, the editors and the reviewers. Any product that may be evaluated in this article, or claim that may be made by its manufacturer, is not guaranteed or endorsed by the publisher.
