# Supplementary material for: Barcode clonal tracking of tissue-resident immune cells in rhesus macaque highlights distinct clonal distribution pattern of tissue NK cells
Source: Front Immunol. 2022 Nov 10;13:994498. doi: 10.3389/fimmu.2022.994498 (PMC9808525; doi:10.3389/fimmu.2022.994498)
Supplement: Supplementary file 1 [file Table_1.docx]

Table 1. Transplantation parameters and follow-up characteristics of animals included in this study.

|  | **JM82** | **ZK22** | **ZJ31** | **ZG66** | **A9E016** |
| --- | --- | --- | --- | --- | --- |
| CD34+ transplant dose (Millions) | 91 | 82 | 23 | 48 | 23 |
| CD34+ transplant dose/kg (Millions) | 7.2 | 7.2 | 4.1 | 8.5 | 4.3 |
| % GFP+ infused cells | 34% | 31% | 35% | 35% | 25% |
| Infused GFP+ cells (Millions) | 30.6 | 25.2 | 8.0 | 16.7 | 5.75 |
| Follow-up timepoints  (Months post transplantation) | 46,47,48,49 | 45,62,63 | 63,74,75,76 | 105,106 | 4 |

Details of transplantation and the clonal patterns of lineage cells in the PB overtime from animals JM82, ZG66, ZJ31 and ZK22 has been previously reported (Truitt et al., 2019), (Wu et al., 2018), (Koelle et al., 2017), (Wu et al., 2014)

Koelle, S.J., Espinoza, D.A., Wu, C., Xu, J., Lu, R., Li, B., Donahue, R.E., and Dunbar, C.E. (2017). Quantitative stability of hematopoietic stem and progenitor cell clonal output in rhesus macaques receiving transplants. Blood *129*, 1448-1457. 10.1182/blood-2016-07-728691.

Truitt, L.L., Yang, D., Espinoza, D.A., Fan, X., Ram, D.R., Mostrom, M.J., Tran, D., Sprehe, L.M., Reeves, R.K., Donahue, R.E., et al. (2019). Impact of CMV Infection on Natural Killer Cell Clonal Repertoire in CMV-Naive Rhesus Macaques. Front Immunol *10*, 2381. 10.3389/fimmu.2019.02381.

Wu, C., Espinoza, D.A., Koelle, S.J., Yang, D., Truitt, L., Schlums, H., Lafont, B.A., Davidson-Moncada, J.K., Lu, R., Kaur, A., et al. (2018). Clonal expansion and compartmentalized maintenance of rhesus macaque NK cell subsets. Sci Immunol *3*. 10.1126/sciimmunol.aat9781.

Wu, C., Li, B., Lu, R., Koelle, S.J., Yang, Y., Jares, A., Krouse, A.E., Metzger, M., Liang, F., Lore, K., et al. (2014). Clonal tracking of rhesus macaque hematopoiesis highlights a distinct lineage origin for natural killer cells. Cell Stem Cell *14*, 486-499. 10.1016/j.stem.2014.01.020.
