# Supplementary material for: Barcode clonal tracking of tissue-resident immune cells in rhesus macaque highlights distinct clonal distribution pattern of tissue NK cells
Source: Front Immunol. 2022 Nov 10;13:994498. doi: 10.3389/fimmu.2022.994498 (PMC9808525; doi:10.3389/fimmu.2022.994498)
Supplement: Supplementary file 6 [file Presentation_1.pdf]

Supplementary Material

Supplemental Figures:

Figure S1:

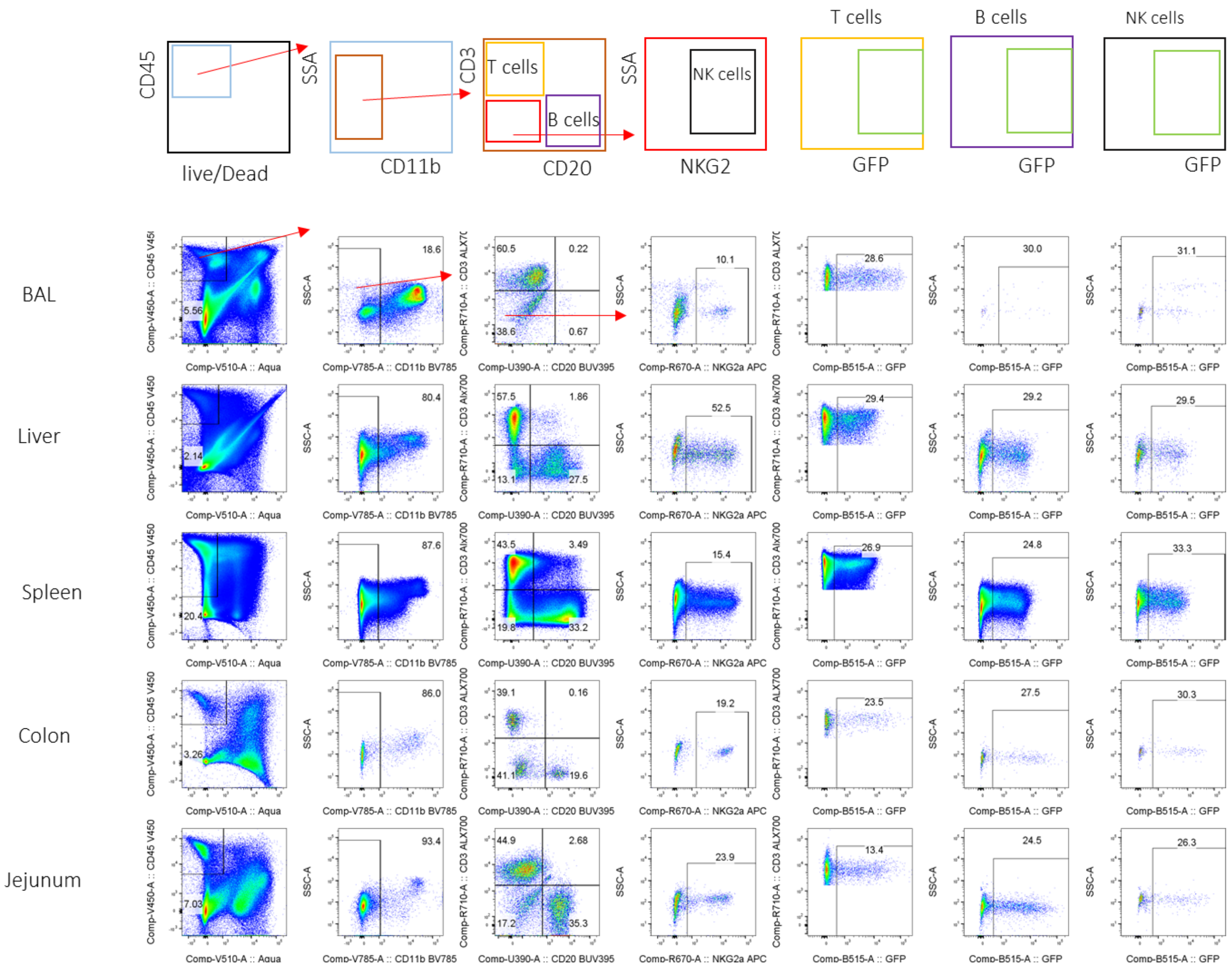

Figure S1: Analysis of GFP expression in tissue samples. Gating schematic is shown in the top row. Representative BAL, Liver, Spleen, Colon and Jejunum samples from animal ZK22 are shown. Cells were gated on live/CD45+ cells, and then the GFP+% cells shown for CD3+ T, CD20+B and CD3-CD20-CD14-NKG2+ NK cells.

Figure S2:

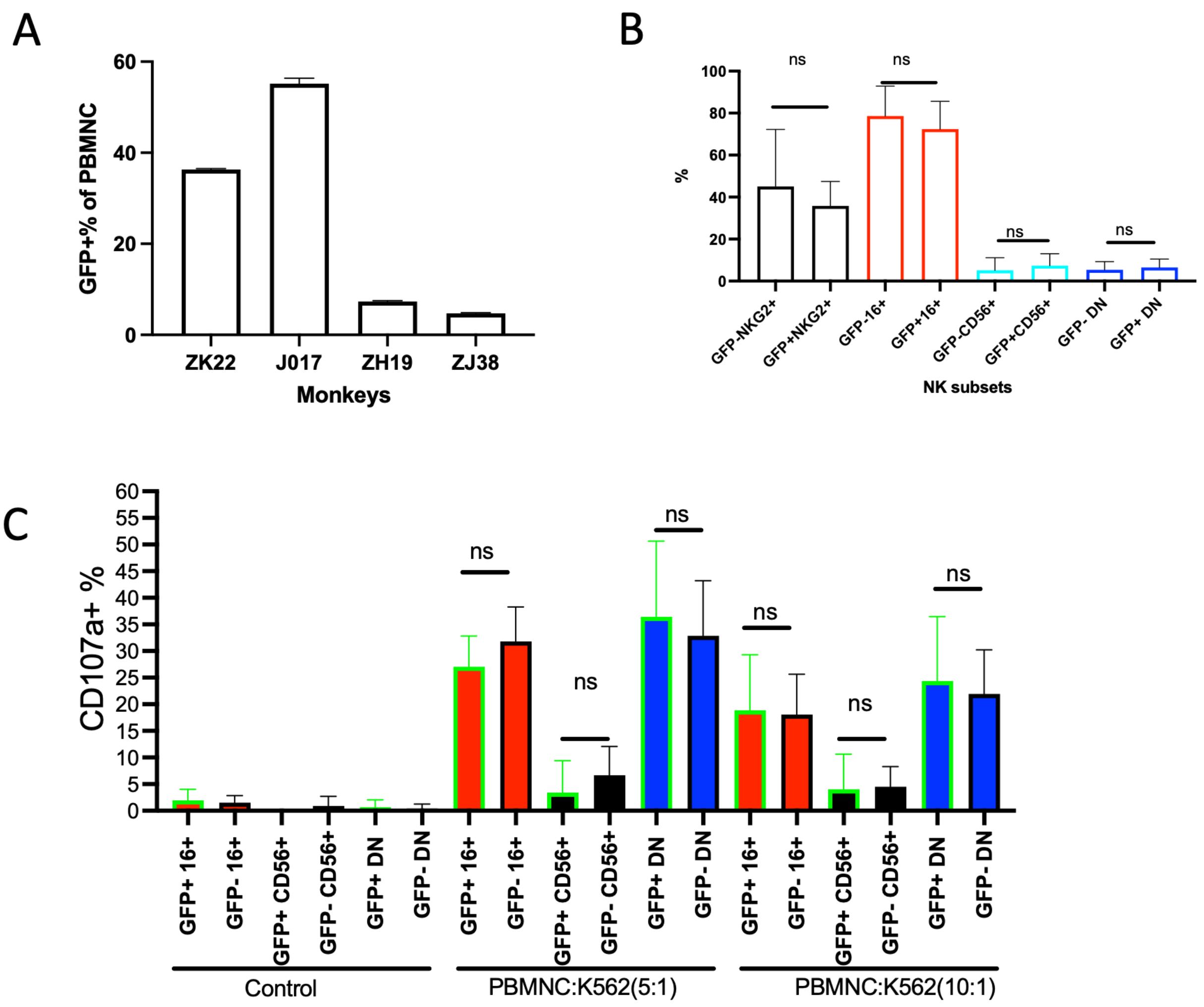

Figure S2: GFP expression does not impact on NK phenotype or degranulation.
(A): % GFP in PBMNCs for the 4 monkey samples analyzed in this figure.
(B): The distribution of immune cell subpopulations in GFP+ versus GFP- cells, showing % bulk NKG2+ within PBMNCs on the left, and CD16+, CD56+ and DN NK moving to the right (n=4).
(C): The percentage of CD107a+ cells in GFP- versus GFP+ NK subsets (CD16+ NK, CD56+ NK, and DN NK) following culture of PBMNCs with/without K562 target cells for 2 hours (n=4).

Figure S3:

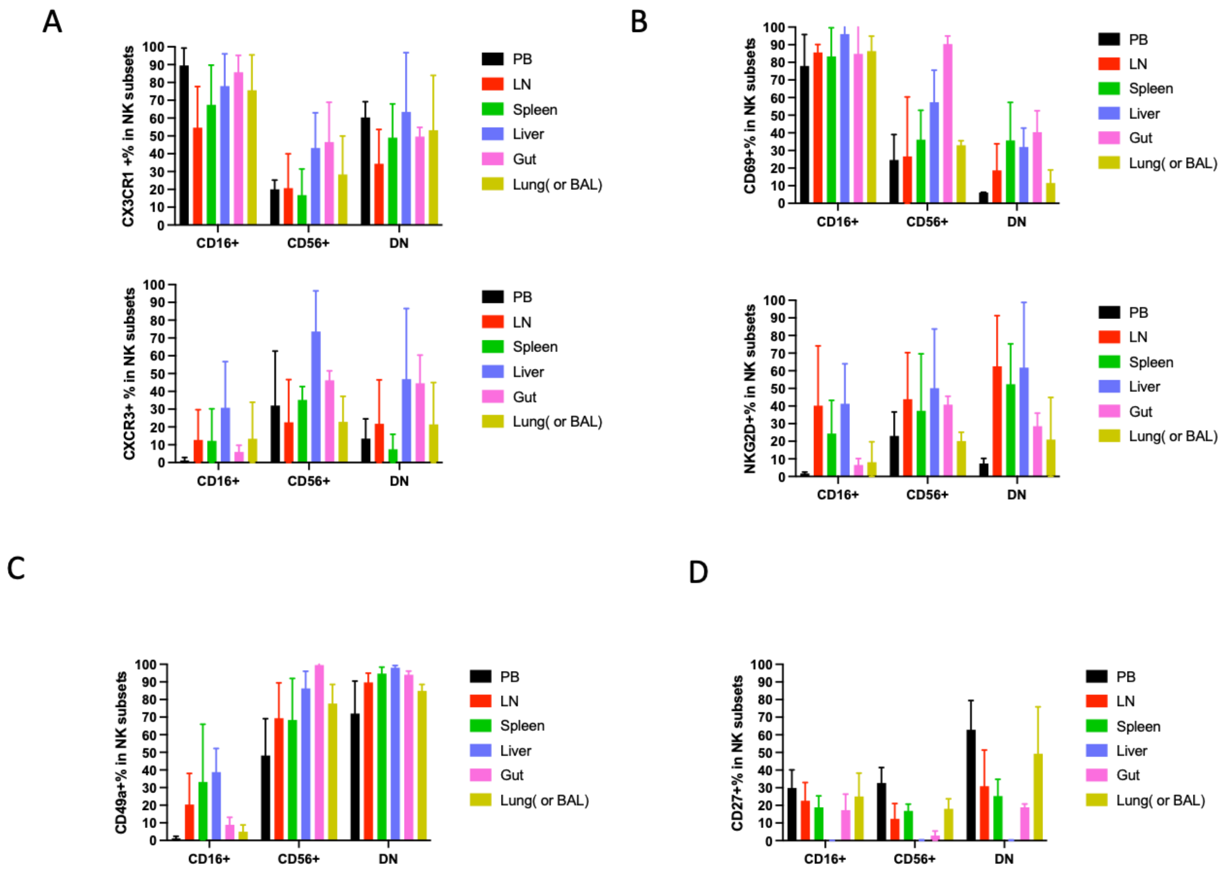

Figure S3: Extended phenotype of NK subpopulations in blood and various tissues

Samples from barcoded monkeys ZJ31, ZG66 and ZJ09 were analyzed, including blood (PB), lymph node (LN), Spleen, Liver, Gut (intestine), lung (ZG66 and ZJ09 only), and BAL from ZJ31. NK cells were gated as CD45+CD3-CD14-CD20-NKG2, and then CD16+, CD56+ and DN NK subsets were analyzed for additional markers separately in panels A-D.

(A): % cells expressing the chemokine receptors CX3XR1 or CXCR3

(B): % cells expressing the activation markers CD69 or NKG2D

(C): % cells expressing CD49a

(D) % cells expressing CD27

Figure S4:

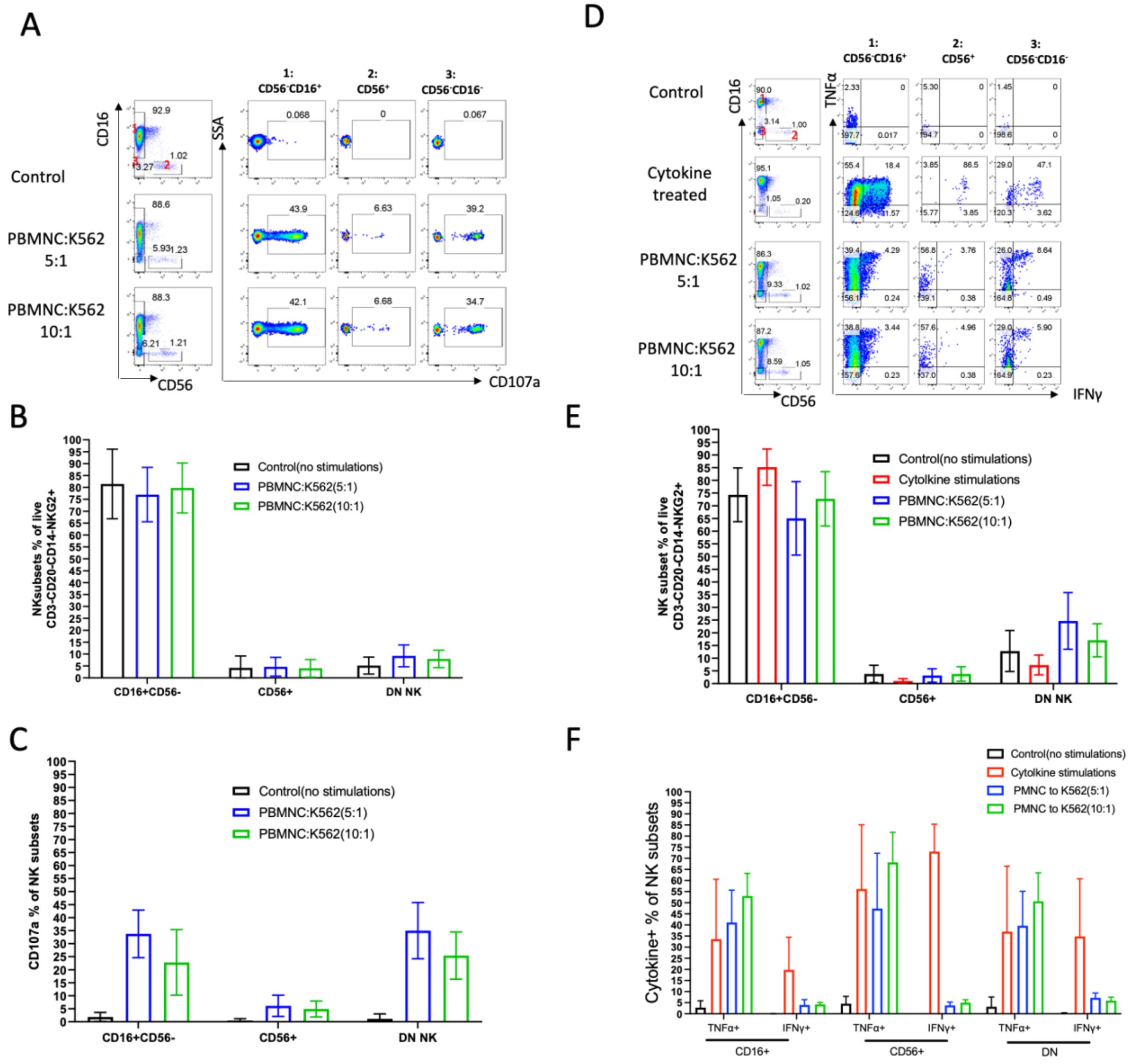

Figure S4: Functional analysis of macaque NK subsets.

(A): Representative flow cytometric plots of CD107a expression on NK cell subsets (animal ZH19) 2 hours post exposure of PBMCs to K562 target cells.

(B): The percentage of each NK subset (CD16+ NK, CD56+ NK, DN NK) within CD3-CD20-CD14-NKG2+ PBMNCs, post exposure of PBMCs to K562 target cells. (n=4 barcoded animals).

(C): The percentage of CD107a+ expression on monkey NK subsets (CD16+ NK, CD56+ NK, and DN NK), post exposure of PBMCs to K562 target cells. (n=4)

(D): Representative flow cytometric plots of IFNγ and TNFα production in CD16+ NK, CD56+ NK, and DN NK subsets following culture of PBMNC with/without K562 cells or cytokine-stimulated with IL15+IL12+IL18.

(E): The percentage of each NK subset following culture of PBMNC with/without K562 cells or IL15+IL12+IL18.(n=4)

(F): The percentage of IFNγ+ and TNFα+ cells in CD16+ NK, CD56+ NK, and DN NK subsets, post culture of PBMNC with/without K562 cells or IL15+IL12+IL18.(n=4)

Figure S5:

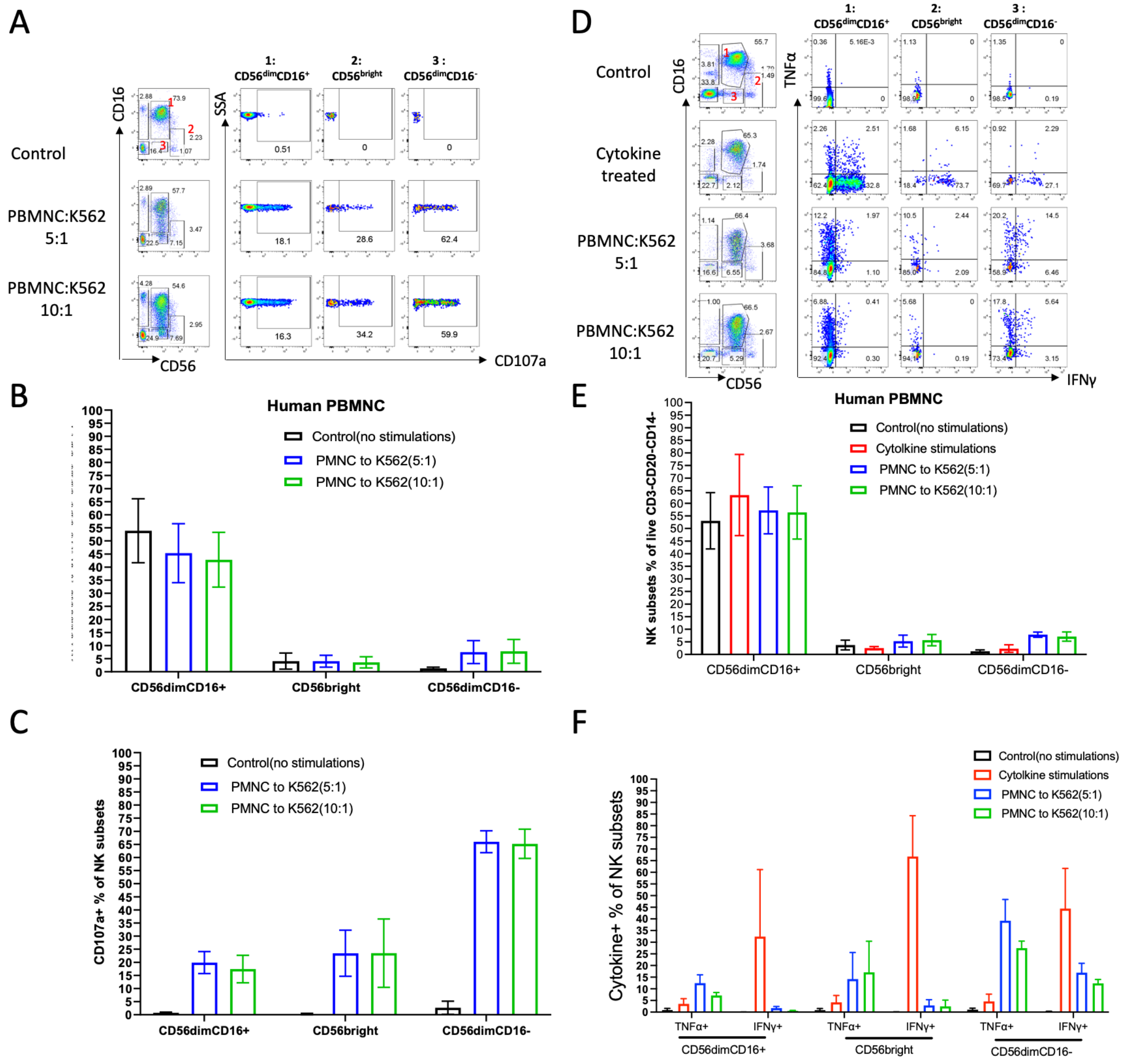

Figure S5: Functional analysis of human NK subsets.

(A): Representative flow cytometric plots of CD107a expression on human NK cell subsets (healthy donor #3) 2 hours post exposure of PBMCs to K562 target cells.

(B): The percentage of each NK subset (CD56dimCD16+ NK, CD56brightCD16+ NK, and CD56dimCD16- NK) within CD3-CD20-CD14- PBMNCs, post culture of PBMNC with /without K562 target cells for 2 hours. (n=3 healthy donors).

(C): The percentage of CD107a+ expression on human NK subsets, post culture the PBMNC with/without K562 target cells for 2 hours. (n=3 healthy donors)

(D): Representative flow cytometric plots of IFNγ and TNFα production in human NK subsets(healthy donor #3) following culture of PBMNC with/without K562 cells or cytokine-stimulated with IL15+IL12+IL18.

(E): The percentage of each NK subset following culture of PBMNC with/without K562 cells or IL15+IL12+IL18.(n=3 healthy donors)

(F): The percentage of IFNγ+ and TNFα+ cells in human NK subsets following culture of PBMNC with/without K562 cells or IL15+IL12+IL18.(n=3 healthy donors)

Figure S6:

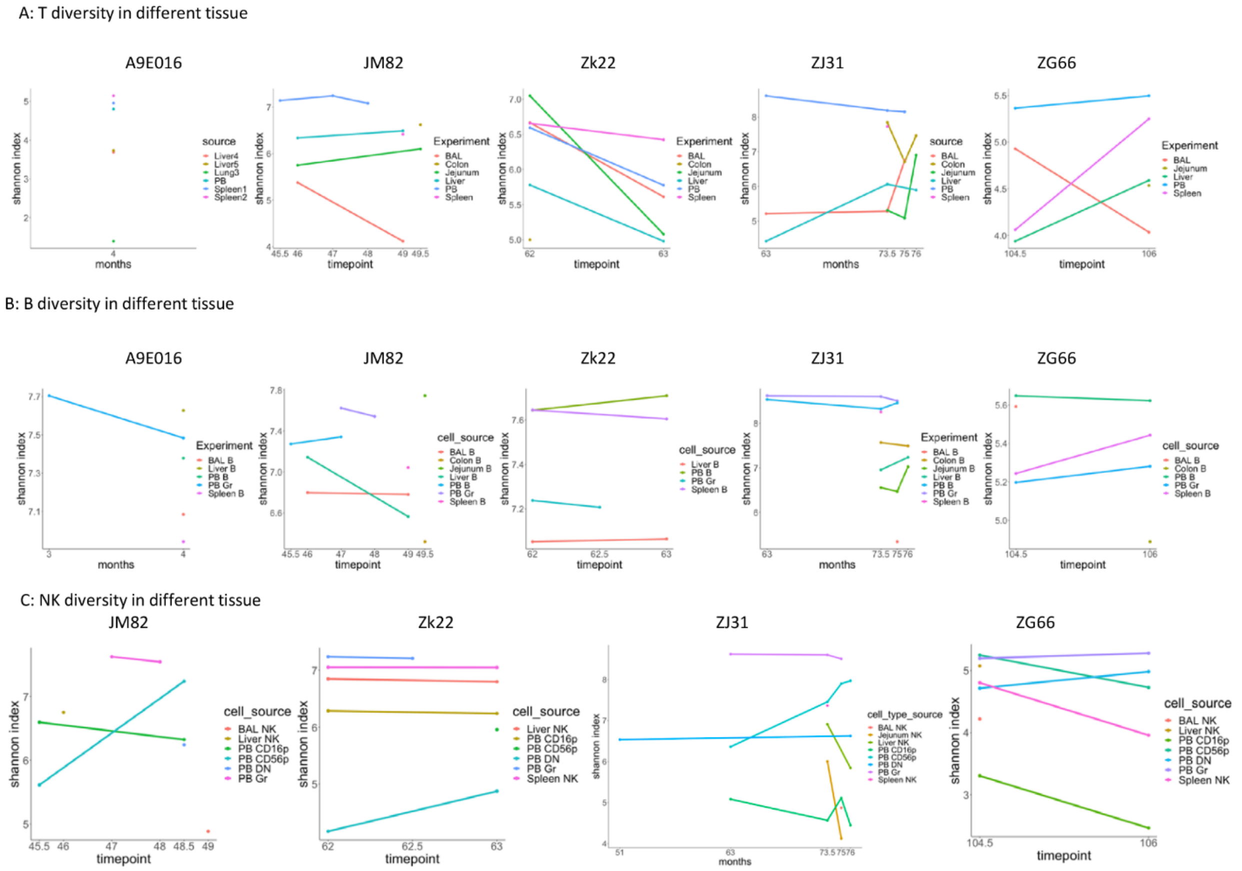

Figure S6: **Shannon diversity indices for all barcode contributions**: (A) T cells, (B) B cells, (C) NKG2+ bulk NK and/or NK subsets CD56−CD16+, CD56+CD16- NK and DN NKs over time in PB and tissues.

Figure S7:

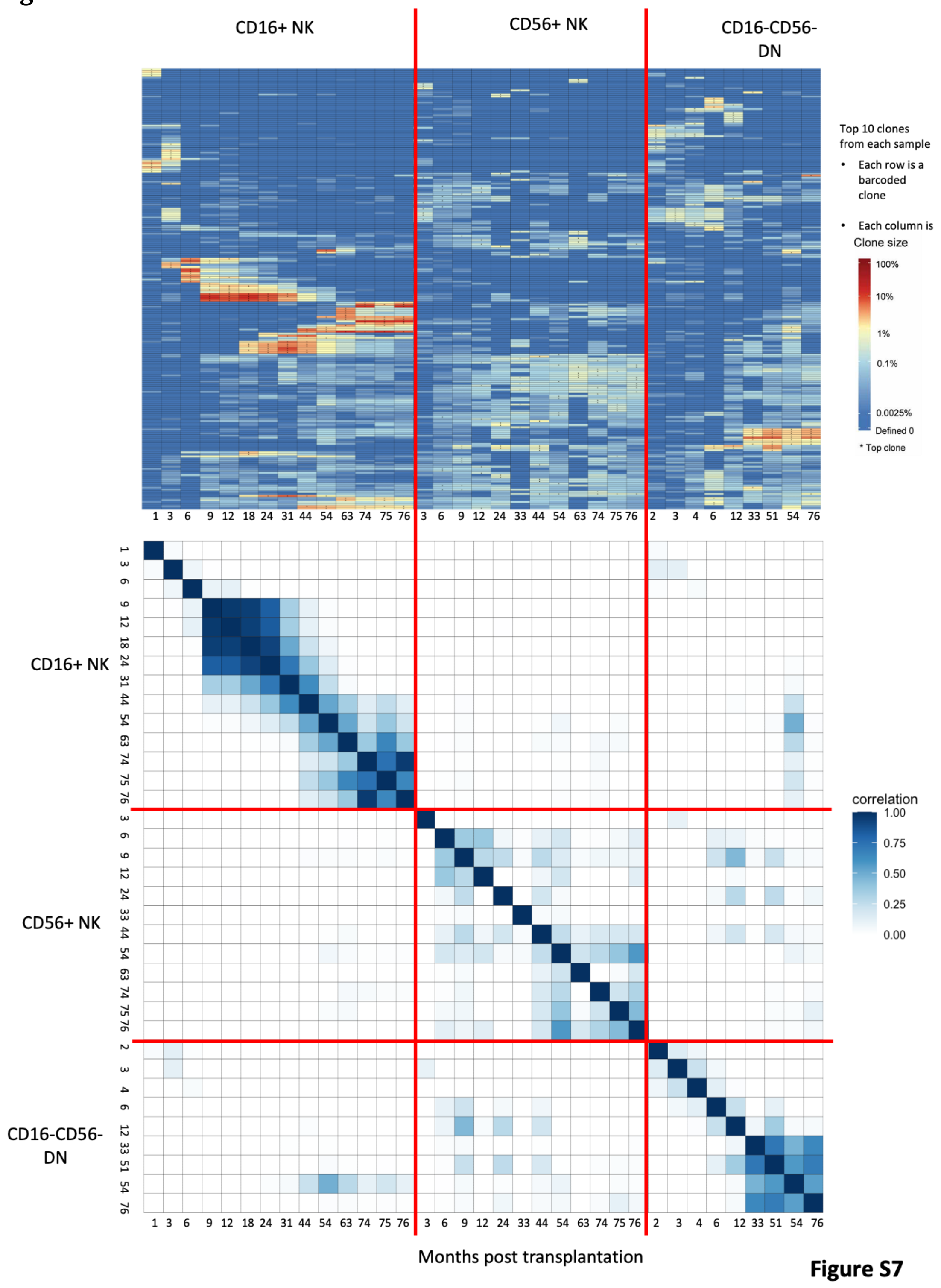

Figure S7: Barcode clonal pattern of NK subsets over time in PB post-transplant in monkey ZJ31.
Top panel is the top 10 clone heat maps of NK subsets overtime in ZJ31 from 1m to 76m.
Bottom panel is the Pearson correlation plot of NK subsets overtime in ZJ31 from 1m to 76m.

Figure S8

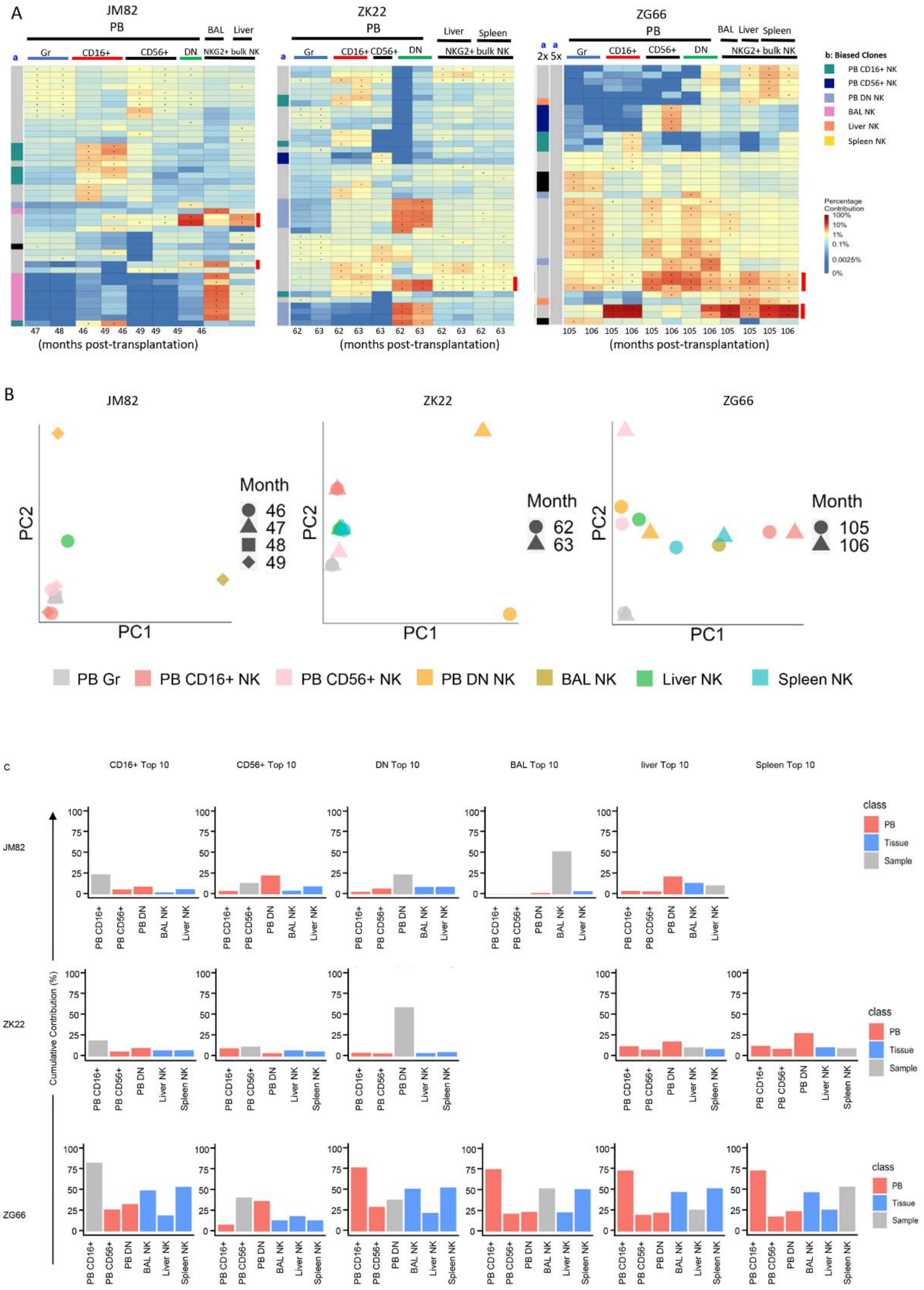

Figure S8: Biased clonal contributions to PB and tissue NK cells (A): The top 10 clones heat maps for JM82, ZK22, and ZG66 PB NK subsets and tissue NKG2+ NK cells. Color blocks in the column "a" on the left of the heatmap shows tissue resident specific biased NKG2+ NK clones. Biased clones are defined as 5x fold change in that tissue compared to all other locations, which indicates that those clones are expanded in the

specific tissue but not in other locations. In ZG66, we also calculated the 2x biased tissue resident expanded NK clones. Color code legends are showed on the right of the panel A.

(B): PCA analysis of the Gr and NK clones from PB and NK clones from tissues in JM82, ZK22, and ZG66. The samples collected from different anatomical locations are represented with different colors, and samples from the same location collected at different time points are denoted using different markers. Samples analyzed are same as the panel A.

(C): The clonal contributions of the aggregate top 10 clones from one NK population to all other populations for JM82, ZK22, and ZG66. In the bar plot, the contribution of the aggregate top 10 clones of the sample to itself is shown in grey, the contribution of the same clones to PB NK subsets is shown in red, and the contribution to the same clones to tissue NK populations is shown in blue. Samples analyzed are same as the panel A.

Figure S9:

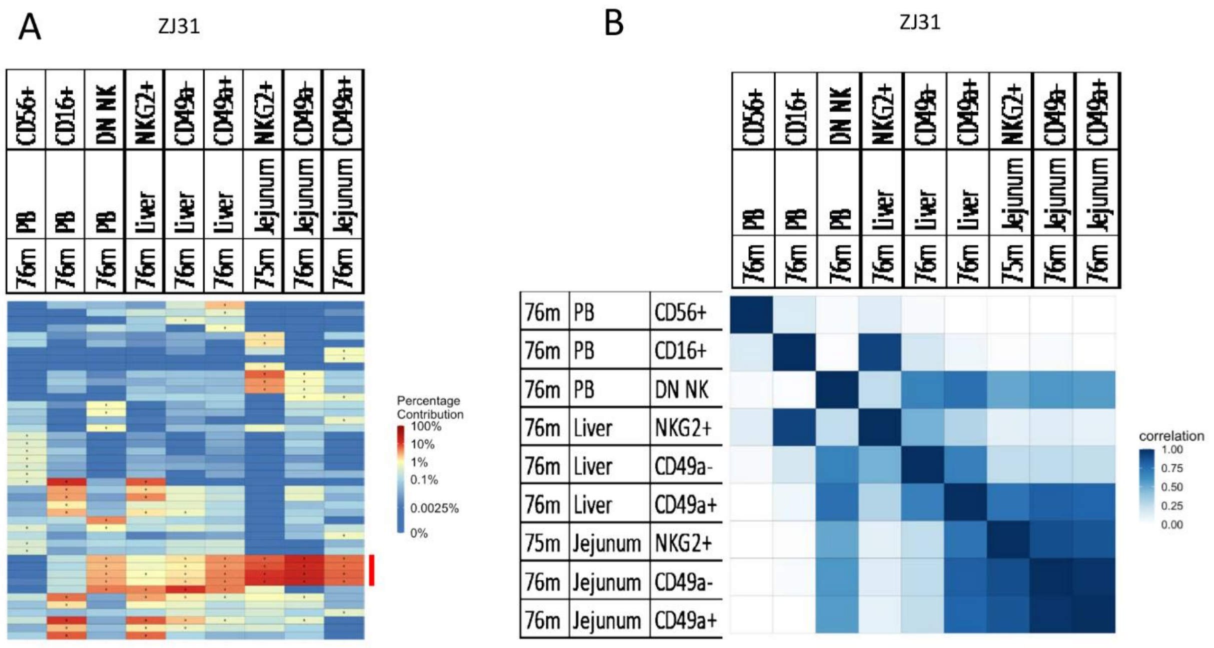

Figure S9: CD49a expression enriches the tissue expanded clones in liver but not in gut tissue.
(A): Top 10 clone heat maps of NK subsets based on CD16, CD56, and CD49a expression in PB, liver and jejunum in monkey ZJ31 at 76m.
(B): Pearson correlation plots of NK subsets based on CD16, CD56, and CD49a expression in PB liver and jejunum in monkey ZJ31 at 76m.

2. Supplemental tables:

1. Table S1: transplantation parameters and follow-up characteristics of animals included in this study
2. Table S2: GFP% for lineage cells in monkeys
3. Table S3: cells count for barcode PCR
4. Table S4: antibodies
5. Table S5: primers
